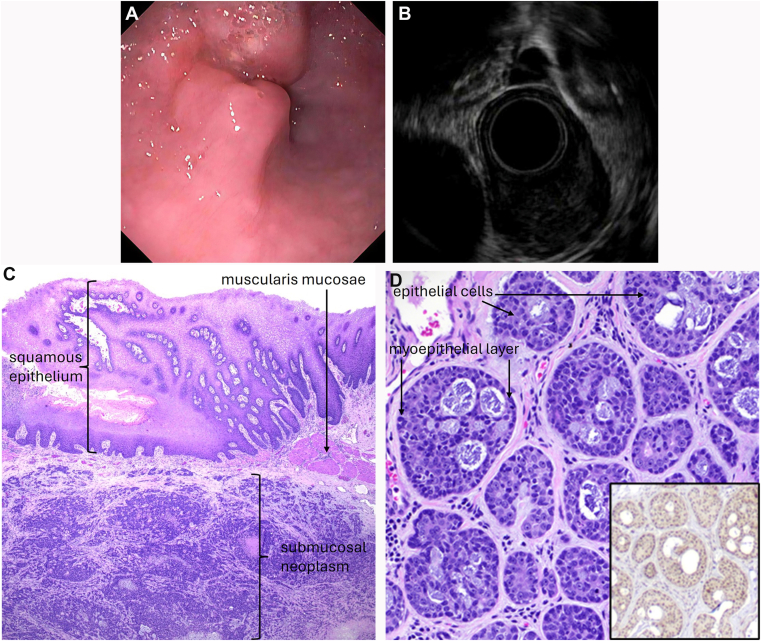# Cribriform Constriction: An Exceedingly Rare Cause of Progressive Dysphagia

**DOI:** 10.1016/j.gastha.2026.101013

**Published:** 2026-05-19

**Authors:** Maria Clara Alves Moreira, Dipti M. Karamchandani, James Michael Mitchell

**Affiliations:** Department of Pathology, University of Texas Southwestern Medical Center, Dallas, Texas

A 64-year-old woman presented with progressive dysphagia and 20-pound weight loss. Esophagogastroduodenoscopy revealed a circumferential stenosis located 28–31 cm from the incisors (Figure A). Endoscopic ultrasonography showed a hypoechoic mass invading the muscularis propria (Figure B). Initial biopsy demonstrated carcinoma, not otherwise specified. Following nutritional optimization, she underwent robot-assisted Ivor Lewis esophagectomy. Macroscopy showed a 2.8 cm submucosal mass with intact overlying mucosa. Microscopic examination revealed a biphasic neoplasm with a prominent cribriform growth pattern characterized by a dual population of inner epithelial and outer myoepithelial cells centered in the submucosa with overlying nondysplastic squamous epithelium (Figures C and D). Myoepithelial cells expressed p40 and calponin; epithelial cells demonstrated cytokeratin and CD117 immunoreactivity. Diffuse nuclear MYB immunoreactivity (Figure D, inset) confirmed the diagnosis of adenoid cystic carcinoma, serving as a surrogate for the characteristic MYB-NFIB fusion. The postoperative course was excellent, with successful transition to oral diet.

Esophageal adenoid cystic carcinoma is exceedingly rare (<0.1% of esophageal malignancies). Accurate diagnosis is vital, as its clinical behavior and surgical management often diverge from the more common squamous or glandular carcinomas. A high index of suspicion, combined with a targeted immunohistochemical workup, is essential for avoiding diagnostic pitfalls and ensuring optimal patient management.

**Figure:**